# The Children of Risk Communication

**DOI:** 10.1177/14034948221110409

**Published:** 2022-07-10

**Authors:** Leena Paakkari, Orkan Okan

**Affiliations:** 1 University of Jyväskylä, Faculty of Sport and Health Sciences, Research Center for Health Promotion, University of Jyväskylä, Finland; 2 Department of Sport and Health Sciences, Technical University Munich, Germany

**Keywords:** Adolescents, risk communication, COVID-19, prevention, health promotion

## Abstract

For the management of the COVID-19 pandemic, risk communication has been a much-needed preventive and educative action to support citizens – including children – to adopt preventive and health protective measures. However, the COVID-19 pandemic is not the only health concern at hand that has raised concerns about the health status of children and required disease-preventive strategies. Bearing in mind the mental health problems and learning losses reported during the pandemic, in this commentary, we will argue that by now, it is time to consider critically if there could be more space for positive communication and education, both alongside and as an integral part of risk communication, to help adolescents not just to survive but also to live the fullest possible life now and in the future. Otherwise, the adolescents of COVID-19 could become ‘the children of risk communication’ to the detriment of their health and well-being.

The COVID-19 pandemic has required the public to practise, daily, a wide range of measures for health protection and disease prevention. The various applicable guidelines have been labelled collectively as ‘risk communication’, which refers to the exchange of information on health risks between experts and citizens [1]. The aim has been to support those at risk so that they make informed decisions and act accordingly [1]. In the COVID-19 period, there have been no individuals who would not be at some risk of infection. Indeed, risk communication has rarely been so important, and has never taken place in a regional and global health crisis of this magnitude. Furthermore, due to the association between lifestyle factors (e.g. obesity and smoking) and COVID-19, the pandemic has multiplied communication on these factors, also underlining their roles as major causes of the global disease burden. For adolescents too, this has meant an added emphasis on the preventive strategies set out in risk-related communication. The nature of this communication requires critical evaluation. In this commentary about adolescence, we refer to the life phase between 10 and 19 years old [2], and use ‘a child’ [3] or ‘an adolescent’ as parallel concepts to refer to that age range.

Adolescence has been recognised as a critical period for establishing patterns of adult health [4]. It also represents a period with its own health risks, including substance misuse, mental health problems and obesity. The protection of adolescents’ health and the prevention of risk behaviours is gaining more attention due to immediate influences on adolescents’ current health status, to long-term influences on health as adults and to intergenerational health mobility on health of the next generations. Hence, prevention strategies have become even more important, aimed at supporting adolescents in addressing risk factors and persisting with protective factors.

The need for prevention is usually identified by measuring health and well-being via predetermined indicators. Due to a lack of measures of positive health, the indicators used among adolescents – and subsequent preventive actions – have tended to focus on either the risks of disease or the causes of death or disability [5]. Given that many risks are related to environmental factors, to individuals’ health-related choices and even to biological and cultural factors, *all* adolescents can ultimately be regarded as exposed to a certain level of risk for developing unfavourable health behaviours and poor health.

One crucial question is this: How much preventive and risk-focused communication and education is needed with adolescents in a situation where no immediate risk has yet been identified? Adolescents are typically still fairly healthy. They may thus face a situation where they have to transfer all the ‘risk talk’ into personal meanings, and to ponder the risk for themselves (i.e. to live with uncertainty) [6]. They may find themselves pressured to consider how they should behave if the risks are actualised, including ‘what it means to consider what it means to be in danger of developing an illness’ even though – as is likely to be the case – they have no symptoms [6]. As Lupton [7] has pointed out, if one accepts that people have the right ‘to be warned of the dangers of their risks’, should they not also have the right ‘to *not* be *continually* [emphasis added] informed of the risks they might be taking when engaging in certain actions’?

The focus of health-enhancing actions should include the building of young people’s personal health assets and agency [8], along with the creation of supportive and safe environments to help adolescents ‘develop to their full potential and attain best health’ [9]. This would imply that positive approaches, with a focus on health assets such as health literacy [10] – with attention also to factors such as a sense of coherence and psychological flexibility – would be optimal in building adolescents’ preparedness to face uncertainties, to cope with changes, to avoid unnecessary health anxieties and to empower them in influencing the determinants of their own health, including also during health hazards such as COVID-19. Furthermore, strengthening adolescents’ health literacy could serve as a complementary strategy in seeking to improve the effectiveness of risk communication [11].

Bearing in mind the mental health problems and learning losses reported during the pandemic caused in part by measures such as school closures, it is time to consider critically what kind of efforts and communication strategies would help adolescents not just to survive but also to live the fullest possible life now and in the future. There is an urgent need to find a balance between discourses on health and disease, on the one hand, and assets-based health promotion and disease prevention, on the other. Otherwise, the adolescents of COVID-19 could become ‘the children of risk communication’ to the detriment of their health and well-being.
